# Rational design of synthetic antimicrobial peptides based on the *Escherichia coli* ShoB toxin

**DOI:** 10.1038/s41598-025-98330-3

**Published:** 2025-04-24

**Authors:** Ingvill Pedersen Sæbø, Emma Dyhr, Ida Mathilde Marstein Riisnæs, Henrik Franzyk, Magnar Bjørås, James Alexander Booth, Emily Helgesen

**Affiliations:** 1 Department of Microbiology, University of Oslo and Oslo University Hospital, Rikshospitalet, Oslo Norway; 2 Department of Drug Design and Pharmacology, Faculty of Health and Medical Sciences, University of Copenhagen, Copenhagen, Denmark; 3 Department of Clinical and Molecular Medicine, Norwegian University of Science and Technology and Clinic of Laboratory Medicine, St. Olavs Hospital, Trondheim, Norway

**Keywords:** Synthetic antimicrobial peptides, Antimicrobial resistance, Antibacterial efficacy, Type-I Toxin-Antitoxin systems, Peptide design, Peptide antibiotic development, Drug discovery, Peptides, Peptide delivery, Bacterial infection, Microbiology techniques, Antimicrobials

## Abstract

Antibiotic resistance is an escalating global concern, necessitating the development of novel antibiotics with unique mechanisms of action, and preferably also with a lowered propensity for resistance development. Type-I Toxin-Antitoxin (TA) systems that are ubiquitous in bacterial genomes consist of a genetic toxin element encoding a hydrophobic peptide and an antitoxin element producing an sRNA that inhibits the toxin translation. Although the biological roles of these membrane-associated toxins remain incompletely understood, their inherent lethality upon overexpression suggests a potential as antimicrobial agents. In this study, we explore the ShoB toxin from the *shoB-ohsC* TA system in *Escherichia coli* (*E. coli*) as a basis for designing synthetic antimicrobial peptides for exogenous delivery. We demonstrate that ShoB-derived peptides can retain antimicrobial efficacy when modified into shorter, cationic analogs with enhanced solubility. Our most promising hits exhibit rapid bactericidal action and frequency of resistance within *E. coli* cultures indicate a limited tendency for resistance development. These findings highlight that type-I TA systems constitute a novel source of potential peptide-based antibiotics, thereby offering an alternative largely unexplored strategy to combat antibiotic-resistant bacterial infections.

**Supplementary Information:**

The online version contains supplementary material available at 10.1038/s41598-025-98330-3.

## Introduction

Due to the continuously increasing challenges concerning the emergence and spreading of antimicrobial resistance (AMR) there is a pressing need for new antibiotics, which has been well documented by some of the most authoritative global health organizations^1,2^. AMR also affects economic development - with most profound effects in the poorest countries that typically have a high incidence of AMR^3^. Critically, the pipeline for clinically approved antibiotics comprises only a limited number of entities that target infections caused by high-priority Gram-negative pathogens^4^.

Emergence of resistance to last-resort antibiotics (e.g., colistin and advanced carbapenems like meropenem, imipenem and doripenem) that have a restricted use for treatment of infections with Gram-negative extended drug-resistant (XDR) and pandrug-resistant (PDR) strains is of particular concern^5–7^.

Over the last decades research and development of peptide therapeutics has made substantial progress due to improved methods that ease their production, modification, and analysis^8^. Peptide-based antibiotics have received considerable attention as promising classes of novel potential antibiotics by academia, while the pharmaceutical industry largely has withdrawn from this field due to a lack economic incentives for development of new antibiotics. This is primarily due to restrictions on the use of novel, potential last-resort antibiotics aiming at mitigating AMR development, coupled with the fact that most patients require antibiotic treatment only for short periods^9^.

Most experimental peptide-based antibiotics are derived from antimicrobial peptides (AMPs) involved in the innate host defense systems of all higher organisms, and hence they are often referred to as host-defense peptides (HDPs)^10–12^. These share common attractive features: (i) readily synthesized short sequences (10–40 residues), (ii) fast-acting, and therefore (iii) exhibiting low propensity for inducing rapid AMR development. Nevertheless, there are some important shortcomings of AMPs that need to be addressed to facilitate development of successful AMP-based antibiotics, including their inherent propensity towards enzymatic degradation and risk of exerting off-target toxicity^13^ and immunogenicity^14^.

Type-I Toxin-Antitoxin systems (TAs), first discovered on bacterial plasmids^15^ and later found as homologs and unique variants in bacterial chromosomes, are genetic systems that are abundant in specific bacterial groups^16^. To date, these systems have been identified primarily in Firmicutes and Proteobacteria, with a subset of species so far known to possess them^16^. They are comprised of a genetic toxin element, encoding a short hydrophobic peptide, and an antitoxin element, encoding a short sRNA that inhibits translation into the toxin^17^. Many type-I peptide toxins affect bacterial membranes^17,18^, at least when overexpressed, however, their precise biological functions are not fully elucidated^19^. One possible scenario is that type-I toxins interact with specific partners, however, this has yet to be demonstrated for systems other than that of *ldrD-rdlD*^20^. Importantly, all type-I toxins share a common characteristic feature of being lethal to the toxin-producing bacteria following overexpression. We have previously shown that the toxins from type-I TA systems can be exploited to eradicate bacteria in vivo by using phage systems for their delivery and expression^21^. However, phage-based therapies have several drawbacks, one of them being rapid emergence of resistance against the phage^22^.

In the present work we aimed to explore a type-I TA system toxin as the starting point for development of potent, synthetic AMPs for exogenous delivery. We focused on the toxin ShoB from the *shoB-ohsC* system in *Escherichia coli* and found that the antimicrobial activity of the ShoB peptide may be retained upon truncation and modification with cationic residues or moieties to obtain analogs with improved solubility. Further, it was demonstrated that ShoB-based peptides exhibit rapid bactericidal activity and low propensity for resistance development when tested against *E. coli*. This work emphasizes that type-I TA systems may be recognized as a unique source of potential novel peptide-based antibiotics.

## Results and discussion

Type-I TA systems are abundant in bacterial genomes, and their toxin counterparts have been shown to exert potent killing effects when overexpressed^23^. The toxins therefore constitute interesting starting points for development of unique subclasses of peptide-based potential antibiotics. One major obstacle that obviously may limit their immediate utility is their inherent pronounced hydrophobicity. Thus, a vital question in this context was whether it would be possible to increase solubility whilst retaining antimicrobial activity.

**Fig. 1:**
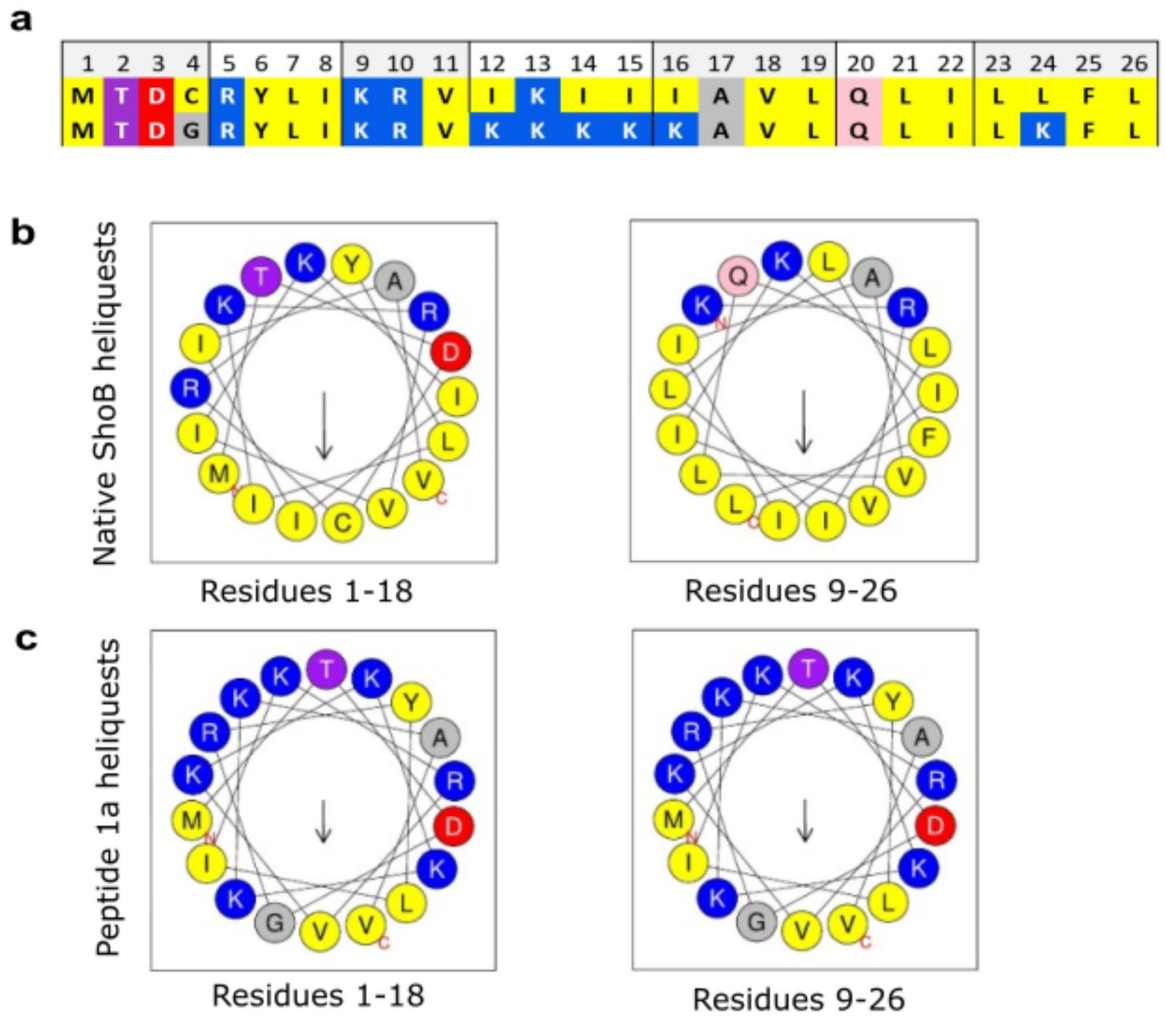
Initial strategy to improve solubility of the ShoB peptide. (**a**) Overview of the amino acid sequence of the native ShoB peptide versus the initially modified peptide **1a**. Color code: yellow, hydrophobic; purple, threonine (T); grey, small residues (glycine (G) and alanine (A)); red, acidic; pink, glutamine (Q); blue, lysine (K) and arginine (R). (**b**) Helical-wheel diagrams (made by Heliquest https://heliquest.ipmc.cnrs.fr/cgi-bin/ComputParamsV2.py) of the native ShoB peptide divided into residues 1–18 (left panel) and 9–26 (right panel). Arrows represent direction and magnitude of the hydrophobic moment. Residues marked C and N (in red) are the C-terminal and N-terminal residues, respectively. Color coding as described above for panel (**a**). (**c**) Helical-wheel diagrams of peptide **1a** divided into residues 1–18 (left panel) and 9–26 (right panel). Arrows represent direction and magnitude of the hydrophobic moment. Residues marked C and N are C-terminal and N-terminal residues of the peptide helix, respectively.

Here, we selected the ShoB peptide from the *shoB-ohsC* type-I system of *E. coli*^24^ as a *proof-of-concept* peptide concerning the possibility to develop type-I toxins into AMP-based potential therapeutics. Expectedly, the chemically synthesized native ShoB peptide toxin (see sequence in Fig. 1a) proved only to be soluble in DMSO - with rapid precipitation when added to bacterial cultures even at low concentrations. Hence, it proved impossible to measure its effect when applied externally to bacteria (data not shown). Therefore, appropriate substitutions in the sequence were made to improve solubility.

Knowing that a high degree of amphipathicity typically is associated with undesirable disruptive effects also on mammalian membranes, we simultaneously aimed at achieving a more even charge distribution along the helix of the peptide (see helical-wheel projections in Fig. 1b and c). Several lysine (K) residues were inserted within the long hydrophobic stretch within the core sequence as well as one in the C-terminal part to give peptide **1a** (Fig. 1a). In addition, the cysteine in the N-terminal part was replaced with glycine to avoid dimerization via spontaneous oxidative formation of disulfide linkages. With these modifications the resulting peptide **1a** possessed reduced amphipathicity (Fig. 1c), and it proved soluble (up to 5 mg/mL in PBS).

Structure prediction using AlphaFold supports that both the native ShoB sequence and peptide **1a** adopt α-helical conformations (Supplementary Figure S1), consistent with the helical-wheel projections shown in Fig. 1b and c. The formation of α-helical conformations has also been shown for other type-I toxins, such as TisB, IbsC, LdrD and AapA1^25–28^. The predicted structural conservation between ShoB and **1a** suggests that the lysine substitutions confer improved solubility while retaining the peptide’s ability to form a helix, an important feature for membrane interactions.

Testing for antibacterial activity in vitro towards a panel of human bacterial isolates revealed promising and broad-spectrum activity of peptide **1a** with Minimal Inhibitory Concentrations (MICs) within the range 8–32 µM depending on the species (Table 1). Killing efficiency (Minimal bactericidal concentration, MBC) was also assessed, and it indicated that peptide **1a** is bactericidal close to its MICs (Table 1).

**Table 1:** MIC and MBC bacterial panel assessment of peptide 1 variants*.

ID	Sequence	MIC / MBC (µM)					
		*E. coli*	*S. aureus*	*K. pneumoniae*	*A. baumannii*	*P. aeruginosa*	*E. faecalis*
**1a**	MTDGRYLIKRVKKKKKAVLQLILKFL	8 / 16	32 / 64	8 / 16	8 / 8	32 / 32	16 / 16
**1b**	MTDGRYLIKRVRKRRRAVLQLRLRFL	32 /16	64 / 64	32 / 64	32 / 32	64 / 64	32 / 16
**1a-N5**	YLIKRVKKKKKAVLQLILKFL	4 / 8	16 / 16	8 / 16	4 / 4	16 / 16	8 / 8
**1a-N9**	RVKKKKKAVLQLILKFL	4 / 8	16 / 16	16 / 8	8 / 8	32 / 32	16 / 16
**1a-N10**	VKKKKKAVLQLILKF	4 / 8	16 / 16	4 / 4	4 / 8	8 / 16	8 / 16
**1a-C3**	MTDGRYLIKRVKKKKKAVLQLIL	16 /32	> 128/>128	> 128/>128	32 / 32	> 128/>128	> 128/>128
**1a-C5**	MTDGRYLIKRVKKKKKAVLQL	> 128/>128	> 128/>128	> 128/>128	> 128/>128	> 128/>128	> 128/>128

*Detection limit:<0.5 µM and > 128 µM.

In an effort to further increase the antimicrobial activity of **1a** we designed a variant with Lys-to-Arg (i.e., K→R) replacements, since this strategy has proved successful for a number of other AMPs^29–31^. Surprisingly, in case of the resulting peptide **1b**, this alteration led to a lowered activity against all bacterial strains in the panel (Table 1). Although Arg can form stronger hydrogen bonds with negatively charged components of the bacterial membrane due to its guanidinium group, it is also more hydrophobic^32^, and hence the corresponding Arg-based analogues may be less soluble in media than the original Lys-containing peptides. Moreover, the bulkier nature of Arg residues may also affect the α-helical structure of the peptide.

**Fig. 2:**
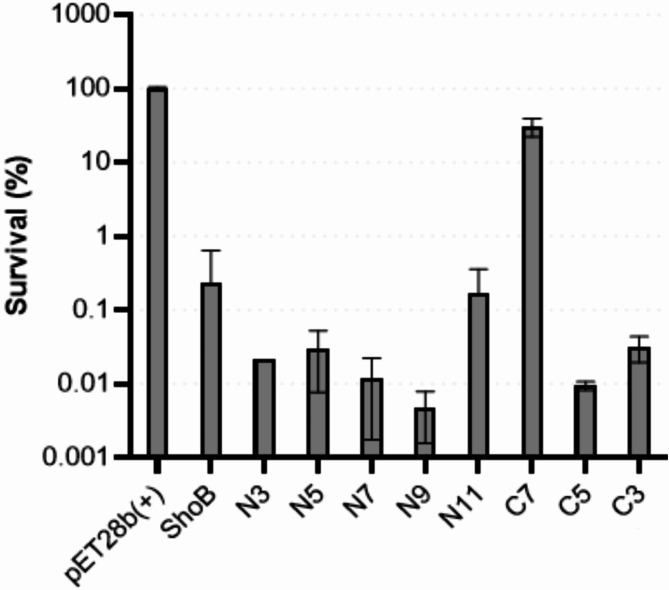
Effect of plasmid-based expression of native and truncated variants of the ShoB peptide. Bar plot showing the percentage of surviving *Escherichia coli* cells (y-axis) after expression of the native, N-terminally truncated or C-terminally truncated variants of the ShoB peptide. The peptides were expressed intracellularly from a plasmid (pET28b(+)) encoding native and truncated variants (see Materials and Methods for details). Standard deviations are indicated in the plot. The experiment was performed in triplicate.

Going forward we instead wished to explore whether shortening of the peptide sequence could be performed while retaining antimicrobial activity. Initially we used a plasmid-based system (pET28b) for bacterial in vivo expression of the native ShoB peptide to obtain indications of the effects of truncations. While intracellular overexpression and external application represent distinct scenarios with different cellular barriers and membrane accessibility, this approach allowed us to systematically probe sequence requirements. The sequence was systematically shortened from either the N- or C-terminus, and the toxicity was assessed by inducing toxin expression and subsequently determining survival (Fig. 2). From these data it was clear that several residues in the N-terminal part could be omitted without affecting intracellular toxicity (i.e., from N1 to N5, N9 or N10), whereas only five residues could be removed in the C-terminal part (Fig. 2; C3 and C5). This indicates that the core and the C-terminal part of ShoB is more important for retained antibacterial properties as compared to the N-terminal part when expressed intracellularly. This is in line with what has been shown also for other toxins from type-I TA systems, such as IbsC^27^ and AapA1 ^26^.

With this as a basis, we tested the truncated synthetic derivatives **1a-C3** and **1a-C5** as well as **1a-N5**, **1a-N9** and **1a-N10** based on the sequence of peptide **1a** to investigate whether the antibacterial effect could be conserved when applied externally. It should be noted that only peptide **1a-N10** was soluble in PBS alone. However, dilution of DMSO stock solutions into media resulted in no precipitation, and the resulting low volumes of DMSO (vehicle) had no growth-inhibitory effect on the bacteria. In line with observations from the plasmid-based system (Fig. 2), the antibacterial activity of N5 and N9 truncated variants (i.e., **1a-N5** and **1a-N9**) was retained or even slightly improved against certain bacterial species (e.g., *E. coli*; Table 1). The **1a-N10** variant, however, exhibited improved activity against the entire bacterial panel. In contrast, truncations in the C-terminal part of peptide **1a** as expected led to analogs with reduced antibacterial activity. The truncation to **1a-C3** resulted in a narrow spectrum of modest activity in *E. coli* and *A. baumannii*, whereas **1a-C5** was devoid of activity (Table 1).

Interestingly, some correlations between the activity during intracellular expression of peptides versus that found for external application were observed (cf. Figure 2; Table 1). This indicates that although the peptides access the membrane from different directions (i.e., from the cytoplasmic side during intracellular expression versus extracellular and/or periplasmic side after external application) the fundamental requirements for their activity appear to be preserved. A study investigating the effect of intracellular expression of the IbsC type-I toxin revealed that modified variants (including truncations and/or mutated residues) that retain toxic activity act via the same mechanisms as that of the wild type peptide when expressed intracellularly, and that this mechanism involves membrane disruption^27^. We do not know whether this is also the case for the ShoB-derived peptides, however, it appears that key structural elements needed for antibacterial activity are similar regardless of the direction of membrane attack. The preserved importance of core and C-terminal regions appears to reflect domains critical for membrane interactions (e.g., folding at or within the membrane) both for attack on the inner or outer leaflet of the cytoplasmic membrane. The small size of the truncated peptides appears to confer sufficient conformational flexibility to achieve their active configuration whether expressed intracellularly or applied externally.

Although the plasmid-based assay (Fig. 2) indicated that continued truncation (e.g., to the N11 ShoB variant) resulted in a loss of activity, further truncation might still provide analogues retaining activity, considering that **1a** and its synthetic analogues in fact are significantly modified versions of the native ShoB peptide. Initially, an **1a-N12** variant, having the original Leu in position 24 (instead of Lys) as in the wild-type ShoB sequence was synthesized (to give peptide **2a-N12**). This peptide had no antibacterial activity (Supplementary Table S1). In an attempt to restore activity, we next sequentially replaced N-terminal lysines with the isoleucines from the original ShoB sequence (to give **2b-N12–2d-N12**; Supplementary Table S1). We also tested several other analogues displaying modifications within this sequence (i.e., **2e-N12–2j-N12**). However, this strategy proved unsuccessful.

We revisited the promising peptide **1a-N10** to explore the effects of K→R substitutions, resulting in analog **3a-N10**, but similar to **1b** this alteration conferred a reduction in activity (Supplementary Table S2). Attaching an Arg_4_ block at the C-terminus, while keeping the original valine and lysine residues at the N-terminal part, led to improved activity of the resulting peptide **3b-N10** (as compared to that of **3a-N10**). This enhancement was evident in *P. aeruginosa*, *S. aureus* and *E. faecalis*, but there was reduced activity in *E. coli*, *K. pneumoniae* and *A. baumannii* (cf. Table 1 and S2).

Next, we examined the insertion of an ultrashort polyethylene glycol residue (i.e., PEG2 = eg1 = -NH-[(CH2)_2_O]_2_-CH_2_-(C = O)-) at different positions: centrally (**3c-N10**), near the N-terminus (**3d-N10**) or near the C-terminus (**3e-N10**), aiming to disrupt parts of the helix or other stable conformation in **1a-N10** (Supplementary Table S2). All three PEG2-modified peptides exhibited reduced activity as compared to **1a-N10**, with the most significant activity loss occurring from helix disruption in the hydrophobic C-terminal part. Notably, **3b-N10** to **3d-N10** retained similar activity against *E. coli*, while **3b-N10** and **3c-N10** were equipotent in *K. pneumoniae*, and **3c-N10** and **3d-N10** had slightly lowered activity (as compared to that of **3a-N10**) in *A. baumannii*.

**Fig. 3:**
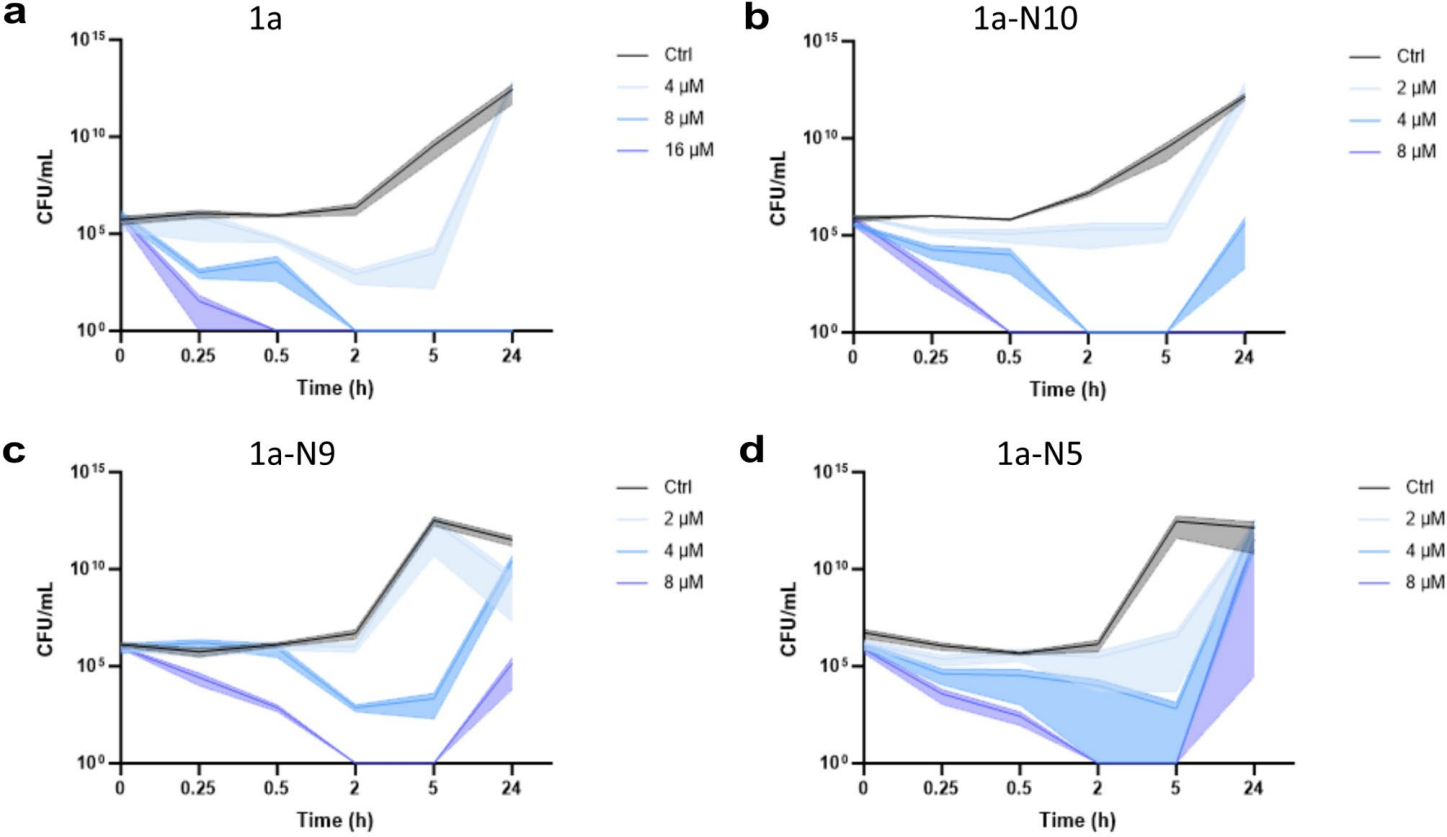
Time-kill kinetics assessment of best peptide hits. Graphs showing the colony-forming units per mL (CFU/mL; y-axis) of *E. coli* ATCC 25922 after incubation with peptide **1a** (**a**), **1a-N10** (**b**), **1a-N9** (**c**), and **1a-N5** (**d**) for 0, 0.25, 0.5, 2, 5 and 24 h (x-axis). The peptide concentrations used are indicated by color coding of the respective graphs, while standard deviations are shown by shaded areas. The limit of bacterial detection in this assay was 100 CFU/mL (see Materials and Methods).

After the above design of multiple analogs from the original ShoB peptide, we characterized the activity profiles of the most promising peptides in more detail. From the MIC and MBC in vitro results it was clear that peptides **1a**, **1a-N5**, **1a-N9** and **1a-N10** exhibited the broadest and most potent antibacterial properties towards the chosen panel of bacteria. As the rate of killing is an important factor for linear peptides with limited enzymatic stability, we decided to conduct kinetics studies of the bactericidal effect via time-kill assays. *E. coli* was chosen for this assay, since the original ShoB sequence used in this context originated from this species, which also appeared to be most sensitive to these peptides (see Table 1). The number of colony-forming units per mL (i.e., CFU/mL) was assessed after different incubation times and exposure to varying concentrations of the respective peptides. The results showed that peptides **1a** and **1a-N10** exhibited the fastest bactericidal activity (Fig. 3a and b), with CFU/ml below the detection limit after 0.5 h at the MBC. For peptides **1a-N9** and **1a-N5** the CFU counts were below the detection limit after 2 h, however, a tendency for regrowth was observed (Fig. 3c and d). The observed regrowth after initial killing could be explained by two distinct mechanisms. First, this phenomenon might indicate selection of suppressor mutations arising under the strong selective pressure of the peptides. Such suppressors could confer varying degree of peptide tolerance through different adaptive mechanisms, explaining the variable regrowth patterns observed in our time-kill assays. Alternatively, regrowth might reflect incomplete sterilization despite CFU counts being below the detection limit. The detection limit in our setup is 100 CFU/mL, meaning that a small population of viable bacteria could remain undetected yet capable of regenerating the population. In this scenario, the observed increase in CFU/mL after 2 h may result from a combination of surviving bacteria and diminished peptide activity due to enzymatic degradation, particularly for peptides **1a-N5** and **1a-N9**. As the peptide concentration decreases over time due to degradation, it may no longer retain sufficient antimicrobial pressure to suppress bacterial growth.

As already discussed, linear all-L peptides (devoid of unnatural residues) are prone to enzymatic degradation. Therefore, our next objective was to increase the stability of the most promising peptides (i.e., **1a** and **1a-N10**) by examining the effect of replacing some or all residues with D-amino acids. Since peptide **1a** is quite long (26 amino acids), we converted the C-terminal part of the peptide (i.e., residues 14–26) into an all-D moiety (to give **D-1a**; Table 2), as this part appeared vital for activity (Table 1; Fig. 2). For peptide **1a-N10** the corresponding all-D form **D-1a-N10** was examined (Table 2).

**Table 2:** Assessment of MIC and MBC of D-form variants (of 1a and 1a-N10-D) in a panel of bacteria*.

ID	Sequence	MIC/MBC (µM)					
		*E. coli*	*S. aureus*	*K. pneumoniae*	*A. baumannii*	*P. aeruginosa*	*E. faecalis*
**D-1a**	MTDGRYLIKRVKK *kkkavlqlilkfl*	16/8	32/16	16/16	32/16	16/16	8/16
**D-1a-N10**	*vkkkkkavlqlilkf*	2/2	2/2	2/2	8/8	8/16	2/4

*Detection limit:<0.5 µM and > 128 µM.

Upon assessment of antibacterial activity, we found that the MIC of **D-1a** as compared to its L-form was increased 2- to 4-fold against *E. coli*, *K. pneumoniae*, *A. baumannii*, *P. aeruginosa* and *E. faecalis*, while it remained unchanged for *S. aureus* (Tables 1 and 2). On the other hand, peptide **D-1a-N10** exhibited unchanged activity (as compared to that of the parent **1a-N10**) in *P. aeruginosa*, 2-fold improved MICs against *E. coli* and *K. pneumoniae*, while 4-fold and 8- fold lowered MICs were seen in *E. faecalis* and *S. aureus*, respectively. In contrast, a slightly reduced potency against *A. baumannii* was found for peptide **D-1a-N10**.

**Fig. 4:**
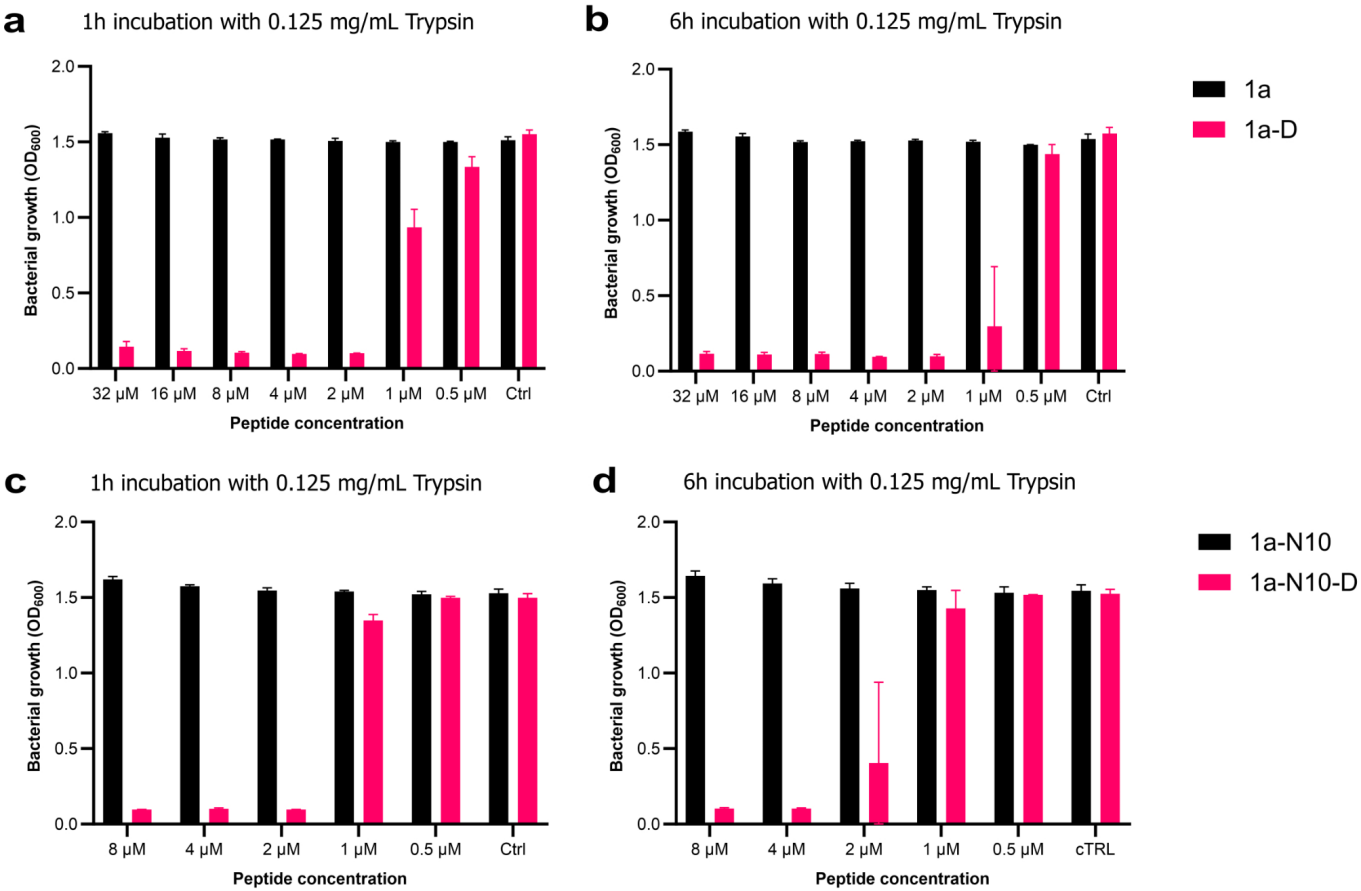
Effect of protease treatment on antibacterial activity of L- and D-form peptides. Bar plots showing the effect of a pre-incubation step, in which peptides **1a**, **D-1a**, **1a-N10** or **D-1a-N10** were treated with 0.125 mg/mL trypsin for 1 h (**a**, **c**) or 6 h (**b**, **d**), prior to testing in a growth inhibition assay with *E. coli* ATCC 25922. The optical density (OD; y-axis) of the *E. coli* cultures was assessed after exposure for 20–23 h to trypsin-treated peptides at the indicated concentrations (x-axis). The experiment was performed in triplicate, and standard deviations are indicated in the plots.

Moving forward, we next tested the proteolytic stability of the D-amino acid-containing variants versus the original all-L forms. The digestive enzyme trypsin was chosen for this assay, as it cleaves at the carboxyl side of Arg and Lys residues which are abundant in our sequences. The peptides were preincubated with 0.125 mg/mL trypsin for 1–6 h, and the ability of the hydrolysis mixtures (containing bot intact AMP and fragments thereof) to inhibit growth of *E. coli* was subsequently assessed. It can be clearly seen that even partial incorporation of D-amino acids conferred retained antibacterial activity even after protease treatment, while the all-L forms appeared to be degraded to inactive fragments after trypsination for 1 h (see Fig. 4). Surprisingly, the apparent MIC of **D-1a** was reduced 8-fold (i.e., from 16 µM to 2 µM) after incubation with trypsin (Fig. 4; panels a and b). This indicates that some favorable degradation within the first 13 residues (i.e., the all-L moiety) occurs, and that this significantly enhances the activity of the resulting degradation product(s). These degradation products may then act via a mechanism similar to that of the truncated peptide variants.

**Fig. 5:**
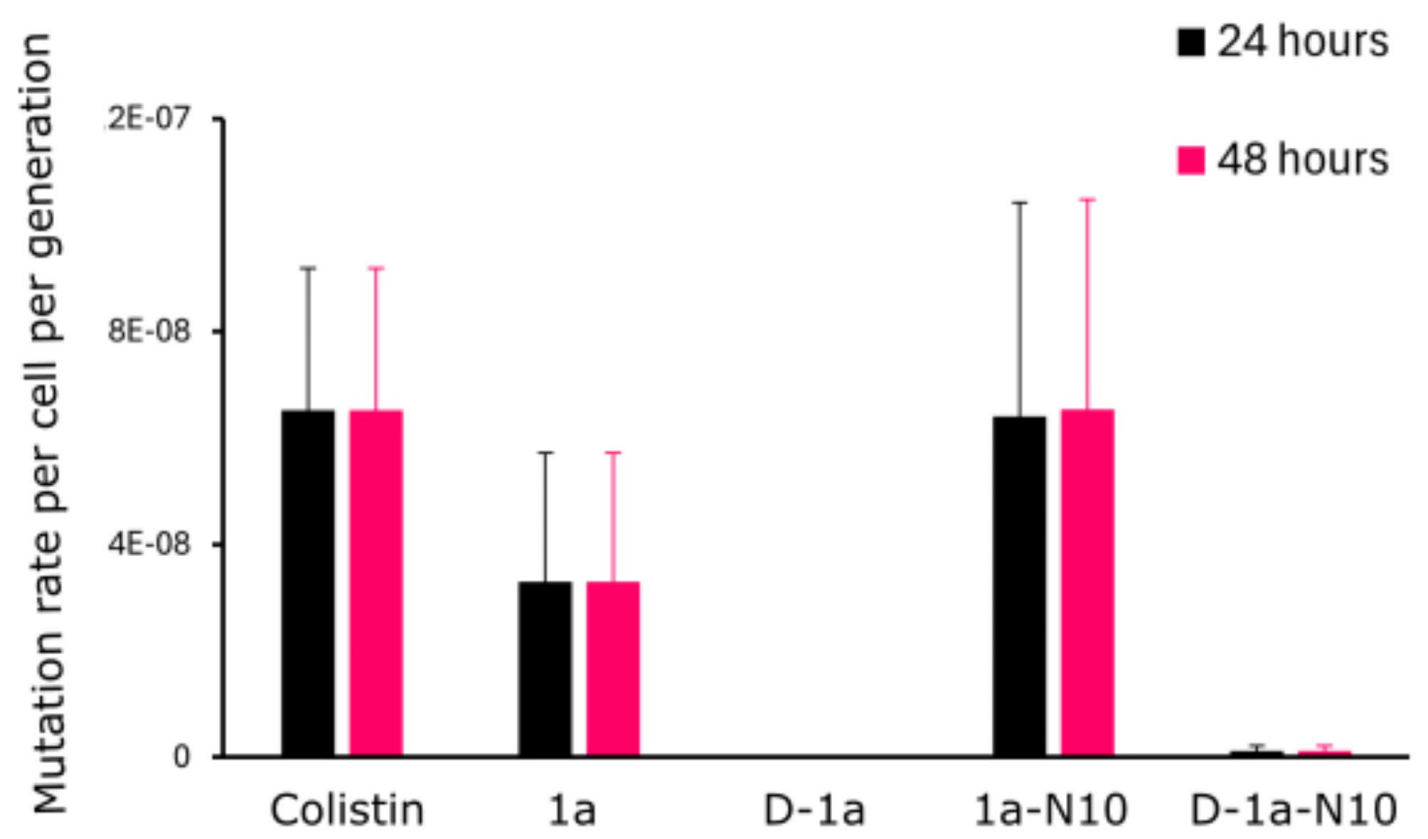
Frequency of resistance (FoR) after treatment with L- and D-form peptide hits for 24 or 48 h. Bar plots showing the mutation rate per cell per generation of *E. coli* ATCC 25922 treated with 4 × MIC of colistin, peptide **1a**, **D-1a**, **1a-N10** or **D-1a-N10** for 24 or 48 h. The values were calculated by using the *p*_*0*_ method (see Materials & Methods for details). The experiments were performed in triplicate, and standard errors of the mean are indicated in the plots.

After confirming that increased proteolytic stability may be conferred by conversion into an all-D or an analog containing an all-D segment (i.e., **D-1a-10 N** and **D-1a**, respectively), we turned to investigate the likelihood of spontaneous resistance occurring under selective pressure exerted by these peptides. We applied an assay based on the *p*_*0*_ method^33,34^, optimized for selection of mutants in liquid medium, to calculate the frequency of resistance (FoR) (see Materials & Methods). Thus, 40 separate cultures of *E. coli* were exposed to each of the top four peptide hits (i.e., **1a**, **D-1a**, **1a-N10** and **D-1a-N10**) at 4 × MIC for 24–48 h, with colistin serving as the reference compound. The results show that exposure to peptides **1a** or **1a-N10** produces low resistance frequencies (i.e., FoRs) comparable to that found for colistin, whereas treatment with the D-amino acid-containing analogs (i.e., **D-1a** and **D-1a-N10**) in fact eliminated mutagenicity, as measured by this method (Fig. 5). As already discussed for the time-kill data of the all-L analogs (Fig. 3), limited enzymatic stability may be the reason for an observed re-growth despite an initial decline in CFU/ml below the detection limit. The complete absence of detectable resistance for D-amino acid-containing variants may reflect one or both of the following distinct mechanisms. First, enhanced proteolytic stability ensures a consistent bactericidal pressure throughout the selection period, thereby preventing survival and subsequent mutation within the bacterial population. Second, the all-D peptides may act via an altered mode of action. Sustained activity due to increased proteolytic stability and/or a membrane-destabilizing mode of action could explain why D-amino acid variants show no detectable resistance development in our assay.

To explore the potential correlation between general membrane destabilization and our FoR data, we conducted a brief preliminary examination of *E. coli* cell morphology following a pulsed exposure to our top four peptide candidates at 10× MIC. Cells were imaged by using phase contrast microscopy after 5 min of exposure. Compared to untreated controls, we observed indications of changes in membrane morphology, particularly for the D-amino acid-containing peptides (**D-1a** and **D-1a-N10**) (Supplementary Figure S2). In contrast, all-L peptide **1a-N10** revealed no explicit membrane defects within the investigated short time period of treatment. These preliminary findings suggest potential differences in membrane interactions among the peptides, which may relate to their proteolytic stability and/or observed variations in FoR. However, additional research using complementary techniques are necessary to conclusively characterize their killing mechanism.

**Fig. 6:**
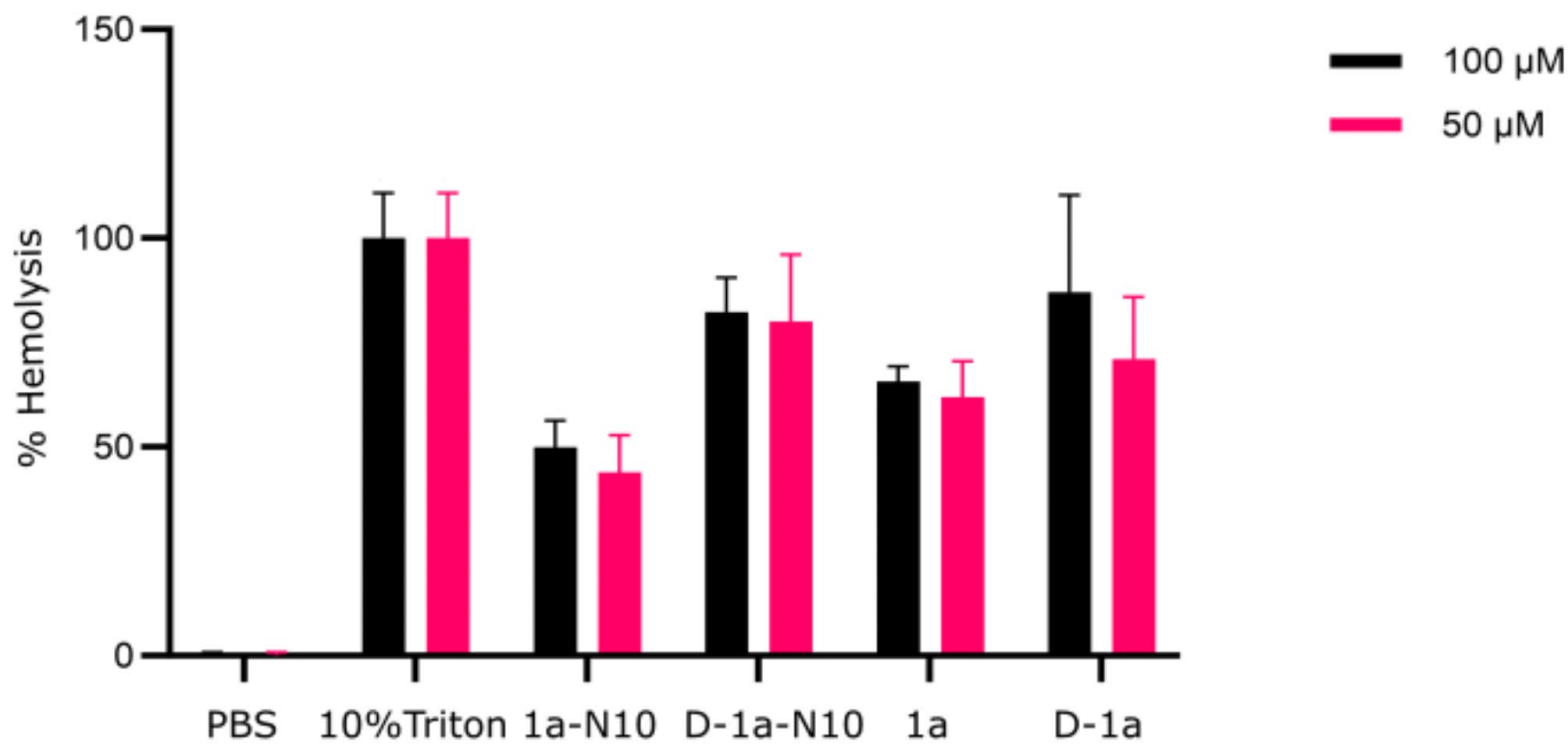
In vitro hemolytic activity of best hits. Bar plots showing the effect of the indicated concentrations of peptides **1a-N10**, **D-1a-N10**, **1a** and **D-1a** on purified erythrocytes by using the hemolysis assay. The hemoglobin content of the supernatant of samples was measured in a spectrophotometer, and the degree of hemolysis (in %, y-axis) was calculated by normalization to negative (PBS-treated cells) and positive (Triton-X-treated cells) control samples. The experiments were conducted with two technical replicates and three biological replicates.

So far, our studies demonstrate that peptide toxins associated with a bacterial toxin-antitoxin system appear to have some potential as starting points for development of potent and stable peptides that do not readily induce AMR development. However, one critical concern is potential off-target toxicity. Therefore, the same four peptides were subjected to an assessment of their ability to disrupt mammalian membranes by using the hemolysis assay^35^. Here, the degree of hemolysis is calculated by normalizing the measured hemoglobin release of peptide-treated erythrocytes relative to that seen for a positive control (giving rise to 100% hemolysis) and a negative control (i.e., providing 0% hemolysis). The erythrocytes were treated with high concentrations of the peptides (i.e., 50 and 100 µM) to estimate an initial safety window. Unfortunately, the present analogs still appear to possess a too high propensity for membrane disruption, and modification of the peptides by incorporation of D-amino acids further aggravated this issue (Fig. 6). Thus, for these toxin-derived antibacterial peptides this toxicity issue remains to be solved.

## Conclusions

In the present study, we have demonstrated that peptides from type-I toxin-antitoxin systems in bacteria indeed can be utilized as starting points in development of potent AMPs. As demonstrated for the ShoB peptide here, the native peptide sequence requires modification with cationic residues or fragments to improve solubility, while truncation will facilitate peptide synthesis. We also find that conversion into analogs displaying D-amino acids confers improved proteolytic stability of the best hits, while also diminishing AMR development in *E. coli*. Unfortunately, the peptides exhibit substantial hemolysis, inferring that future efforts in further optimization of cell selectivity are required. Also, formulation into e.g., nanoparticles may facilitate reduction of hemolytic properties and other potential toxicity and immunogenicity issues.

## Materials and methods

### Peptide synthesis

Peptides **1a**, **1b**, **1a-N5**, **1a-N9**, **1a-C3**, **1a-C5** and **D-1a** were obtained from Genscript Inc. (Piscataway, NJ, USA), while the remaining analogs were synthesized in-house. All starting materials and solvents were purchased from commercial suppliers (Iris Biotech, Germany; Merck, Germany; and Fluorochem, UK) and used without purification. Water for analytical and preparative high-performance liquid chromatography (HPLC) was filtered through a 0.22 μm capsule filter on an Evoqua LaboStar Pro TWF UV system. Preparative HPLC of peptides was performed on either a Phenomenex Luna Omega Polar C18 column (250 × 30 mm; particle size: 5 μm; pore size 100 Å) or a Phenomenex Luna Omega Polar C18 column (250 × 21.2 mm; particle size: 5 μm; pore size 100 Å) by using a Shimadzu Prominence system. Elution was performed with H_2_O-acetonitrile (MeCN) gradients with 0.1% trifluoroacetic acid (TFA) added to the eluents (i.e., A: 5:95 MeCN–H_2_O + 0.1% TFA; B: 95:5 MeCN–H_2_O + 0.1% TFA) with UV detection at λ = 220 nm. Depending on the compound (and column size) gradients of either 0–30% B, 0–50% B, 0–60% B, 20–60% B or 20–50% B over 20 min (flow rate: 20 mL/min; 21.2-mm column) or 30 min (flow rate: 40 mL/min; 30-mm column). The purity of each peptide was determined via analytical HPLC by using a Phenomenex Luna C18 HST column using the same eluents as for the preparative HPLC in a linear gradient from 0 to 60% B during 15 min (flow rate: 0.5 ml/min) and UV detection at 220 nm.

### Microwave-assisted automated synthesis of peptides

Peptides **1a-N10**, **2a-2j**, **3a-3e** and **D-1a-N10** were prepared by Fmoc-based SPPS on either a microwave-assisted CEM™ Liberty Blue synthesizer or on a Gyros Protein Technologies peptide synthesizer. An H-Rink-amide resin from Matrix Innovation (loading 0.50 mmol/g; 0.1 mmol scale; 100–200 mesh) was used as solid support. Coupling of N^α^-Fmoc-protected amino acid building blocks (5.0 equiv for the CEM™ Liberty Blue, and 3.0 equiv for the Gyros Protein Technologies), with acid-labile tBu/Trt/Boc/Pbf side-chain protecting groups, in DMF were performed upon addition of diisopropylcarbodiimide (DIC, 0.5 M in DMF; 5.0 equiv) and ethyl (hydroxyimino)cyanoacetate (OxymaPure^®^, 0.5 M in DMF, 5.0 equiv) in a 0.10 mmol scale. Fmoc-Arg(Pbf)-OH was triple-coupled (for peptides **2i** and **2j**: each time at 75 °C for 10 min; or for peptides **3a-3e**: at room temperature for 30 min followed by 75 °C for 2 min). Other amino acids were each double-coupled at 75 °C for 10 min. For peptides **1a-N10** and **2e-2j** Fmoc-Ile-OH was triple-coupled at 75 °C for 10 min; while PEG2 residues were single-coupled for 10 min at 75 °C. Fmoc deprotection: 20% (v/v) piperidine in DMF (2 × 3 min at room temperature). Side-chain deprotection and cleavage from linker were performed simultaneously with TFA-H_2_O-triisopropylsilane (95:2.5:2.5; 2 × 30 min, each with 5 mL, while shaking at room temperature). The filtrates were collected, and the resin was then eluted with CH_2_Cl_2_ (2 mL). The combined filtrates and CH_2_Cl_2_ were combined and concentrated *in vacuo*, and then co-evaporated with toluene (2 × 5 mL). The crude product was purified by preparative HPLC, and the pure fractions were concentrated and verified by MS, while purity was determined by analytical HPLC.

### Bacterial strains

The panel of bacterial reference strains used in this work for assessment of synthetic peptide activity originated from the American Type Culture Collection (ATCC) and was obtained from the Diagnostic section at the Department of Microbiology, Oslo University Hospital. The strains used were as follows: *E. coli* ATCC 25922, *K. pneumoniae* ATCC 13883, *A. baumannii* ATCC 17978, *S. aureus* ATCC 29213, and *E. faecalis* ATCC 29212.

### Plasmid-based peptide truncation assay

The vectors used for expression of native and truncated variants of the ShoB peptide were produced by Genscript Inc. (Piscataway, NJ, USA), using pET28b (+) as backbone. The vectors were transformed into electrocompetent *E. coli* (ER2566) the same day as assay execution to avoid emergence of suppressor mutations (due to leakage of toxin expression from the vectors). The transformed cells were immediately grown in Luria-Bertani Broth (LB) containing kanamycin (50 µg/mL) for 6 h before dilution to optical density (OD_600_) 0.5. The cultures were subsequently further diluted 10-, 100-, 1000-, 10,000- and 100,000-fold, and each dilution was plated in triplicate onto LB-agar containing kanamycin (50 µg/mL) and 0.2 mM isopropyl β-D-1-thiogalactopyranoside (IPTG) for induction of peptide expression. The plates were incubated at 37^o^C overnight and colonies were counted the day after. The number of colonies were compared with that of the strain containing the empty vector. The experiment was repeated 3 times.

### Preparation of inoculum for susceptibility assays

Bacterial strains were plated on cation-adjusted Mueller-Hinton (MH II) agar plates and incubated at 37^o^C overnight. The day after single colonies were picked and resuspended in sterile-filtered saline (0.9%). The OD of each bacterial suspension was adjusted to McFarland standard 0.5 using a spectrophotometer (UV-1800, Shimadzu), and the suspension further diluted 1:100 in MH II broth, corresponding to 10^6^ colony forming units (CFU) per mL. The inoculum suspension was used for MIC, MBC, time-kill and protease stability assessments as described below. To ensure correct inoculum sizes, 10 µL of the bacterial suspensions were diluted 2000-fold in sterile-filtered saline (0.9%) and 100 µL plated onto MH II agar plates. After overnight incubation at 37^o^C, colonies were counted. If found to be outside the acceptable number (20–80 colonies per plate corresponding to ∼10^6^ CFU/mL), results from further analyses were discarded.

### Minimum inhibitory concentration (MIC)

The peptides were serially diluted (two-fold) directly in 96-well plates (Greiner, MICROPLATE, 96 WELL, PP, U-BOTTOM, NATURAL, 650261) containing MH II broth, corresponding to concentrations in the range 256–0.5 µM. An equal volume of bacterial suspension was added to each well, resulting in a final inoculum of 5 × 10^5^ CFU/mL and final peptide concentrations of 128–0.25 µM. The 96-well plates were incubated for 18–24 h at 37^o^C, after which the OD of each well was measured at 600 nm in a plate reader (Victor Nivo, Perkin-Elmer). The minimum inhibitory concentration (MIC) was estimated as the lowest concentration of peptide that produced OD_600_ measurements similar to the control wells containing no bacteria. The experiments were performed in triplicate. Two-fold variations in MIC values were often observed between replicates. The highest number was reported in Tables.

### Minimum bactericidal concentration (MBC)

In order to assess the minimum bactericidal concentration (MBC) of peptides, 5 µL from each well of the 96-well plate used for MIC analysis was plated onto MH II agar and incubated at 37^o^C overnight. The day after, the agar plates were visually inspected to identify the lowest concentration of peptide that exhibited no regrowth of bacteria (i.e. no visible colonies). The experiments were performed in triplicate. Two-fold variations in MIC values were often observed between replicates. The highest number was reported in Tables.

### Time-kill experiments

Four Eppendorf tubes were set up containing 0.5 mL of *E. coli* inoculum, prepared as described above. To the tubes were added peptides corresponding to final concentrations of ½×MIC, MIC and 2×MIC (see Fig. 3). One tube served as growth control (no added peptide). Then, 50 µL of culture were sampled from the tubes at timepoints 0, 0.25, 0.5, 2, 5 and 24 h. The samples were serially diluted in saline to reach 10^− 5^ CFU/mL. 10 µl of each serial dilution was plated onto MH II agar plates in duplicate, giving a detection limit of 100 CFU/mL. The day after, CFU/mL was calculated based on colony counts and dilution factor. The experiment was performed in triplicate.

### Protease stability assessment

Trypsin solution was prepared by dissolution of Trypsin (Gibco™ Trypsin-EDTA (0.05%), phenol red, 11580626) in PBS (pH 7) to obtain a final concentration of 0.25 mg/mL. Peptides were two-fold serially diluted in trypsin solution in 96-well plates (Greiner, MICROPLATE, 96 WELL, PP, U-BOTTOM, NATURAL, 650261) and incubated for 1–6 h at 37^o^C. The plates were subsequently heated at 60^o^C for 15 min to terminate the enzyme reaction. Bacterial susceptibility was next assessed by adding an equal volume of *E. coli* ATCC 25922 inoculum to the wells and following the protocol of the above described MIC assay.

### Frequency of resistance determination

The method was based on the Fluctuation assay (*p*_*0*_ method; Luria and Delbrück) for determination of mutation rate^33,34^, and optimized for selection of mutants in liquid medium. Colistin sulfate (Thermo Scientific, 15575146) served as control in experimentation. Initially, the MIC of colistin and peptides was verified through broth dilution in 96-well plates (as detailed above). In order to account for potential phenotypic heterogeneity among colonies derived from the same frozen stock, multiple colonies of *E. coli* ATCC 25922 were picked and inoculated in 2 mL MHII broth overnight at 37*°*C with continuous shaking. The following day, aliquots of colistin and peptide solutions were prepared in MHII broth at working concentrations 20 times the MIC. Five 96-well polypropylene plates (Greiner, MICROPLATE, 96 WELL, PP, U-BOTTOM, NATURAL, 650261) were next prepared, one for colistin and one for each of the respective peptides. Then 40 wells per plate were designated for treatment, and added 39 µL MHII broth, 10 µL colistin or peptide working solution (20 x MIC), and 1 µL overnight culture of *E. coli* ATCC 25922, resulting in a final colistin/peptide concentration of 4 × MIC. Two columns in each plate served as growth controls (no colistin/peptide added) and sterility controls (only MHII broth), respectively. The plates were incubated at 37^o^C in a humidified box, and OD was measured at 600 nm using a microplate reader (Victor Nivo, Perkin-Elmer) after 0, 24 and 48 h of incubation. OD_600_ values above 0.5 were defined as growth.

To calculate the viable bacterial counts, the overnight culture was serially diluted 10-fold in MHII, after which 5 µl of the 10^− 3^ – 10^− 9^ dilutions were spotted onto MHII agar plates and incubated overnight at 37^o^C. CFUs were counted the following day to determine CFU/mL of the overnight culture. This value was further divided by 1000 to reflect the actual number of cells in each treated culture. The mutation rate, or frequency of resistance, was calculated using the following formula:

Mutation rate = $$\:-\frac{1}{\text{N}}\times\:\text{l}\text{n}\:{p}_{0}$$

Where mutation rate is defined as the mutation rate per cell per replication cycle (generation), N is the number of viable cells put into each well, and *p*_*0*_ is the proportion of cultures giving rise to no mutants (OD_600_ below 0.5). The experiment was performed in triplicate.

### Determination of hemolytic properties

Blood samples were obtained from a human volunteer using a Vacuette butterfly needle (23 G, blue) attached to a 19 cm tube with a luer adapter (Greiner Bio-One). The blood was drawn from peripheral arm veins and collected in 4 mL Lithium Heparin tubes (Greiner Bio-One). Following phlebotomy, the blood samples were promptly centrifuged at 1700× g for 5 min. After centrifugation, the supernatant was carefully aspirated, and 2 mL of PBS (pH 7) was added. This washing step was repeated thrice or until the supernatant became clear. Subsequently, the remaining pellet was diluted 1:100 in PBS (pH 7) to obtain a 1% erythrocyte suspension. In a 96-well polypropylene PCR plate from VWR, 50 µL of either peptide solution, negative control (PBS, pH 7), or positive control solution (10% Triton X-100) was mixed with 50 µL of the blood sample (1% erythrocyte suspension). The samples were then incubated at 37 °C for 60 min. After the incubation period, the plates were centrifuged again at 1700× g for 5 min. Subsequently, 50 µL of the supernatants were transferred to transparent, flat-bottom 96-well plates (Anicrin). Finally, the absorbance was measured at 405 nm by using a microplate reader (Victor Nivo, Perkin Elmer). The degree of hemolysis was calculated by using the following formula: $\:H\left(\%\right)=\frac{{\text{O}\text{D}}_{test}-{\text{O}\text{D}}_{neg}}{{\text{O}\text{D}}_{pos}-{\text{O}\text{D}}_{neg}}$ × 100%.

## Electronic supplementary material

Below is the link to the electronic supplementary material.

Supplementary Material 1

## Data Availability

The data underlying this article will be shared on reasonable request to the corresponding authors.
