# Supplementary material for: Rational design of synthetic antimicrobial peptides based on the Escherichia coli ShoB toxin
Source: Sci Rep. 2025 Apr 24;15:14354. doi: 10.1038/s41598-025-98330-3 (PMC12022103; doi:10.1038/s41598-025-98330-3)
Supplement: Supplementary file 1 — Supplementary Material 1 [file 41598_2025_98330_MOESM1_ESM.pdf]

SUPPLEMENTARY DATA

Rational design of synthetic antimicrobial peptides based on the *Escherichia coli* ShoB toxin

Ingvill Pedersen Sæbø[1], Emma Dyhr[2], Ida Mathilde Marstein Riisnæs[1], Henrik Franzyk[2], Magnar Bjørås[1,3], James Alexander Booth[1,3*] and Emily Helgesen[1,3*]

[1]Department of Microbiology, University of Oslo and Oslo University Hospital, Rikshospitalet, Oslo, Norway

[2]Department of Drug Design and Pharmacology, Faculty of Health and Medical Sciences, University of Copenhagen, Copenhagen, Denmark

[3]Department of Clinical and Molecular Medicine, Norwegian University of Science and Technology and Clinic of Laboratory Medicine, St. Olavs Hospital, Trondheim, Norway.

[*]Corresponding Authors (james.booth@ntnu.no & emily.helgesen@ous-research.no)

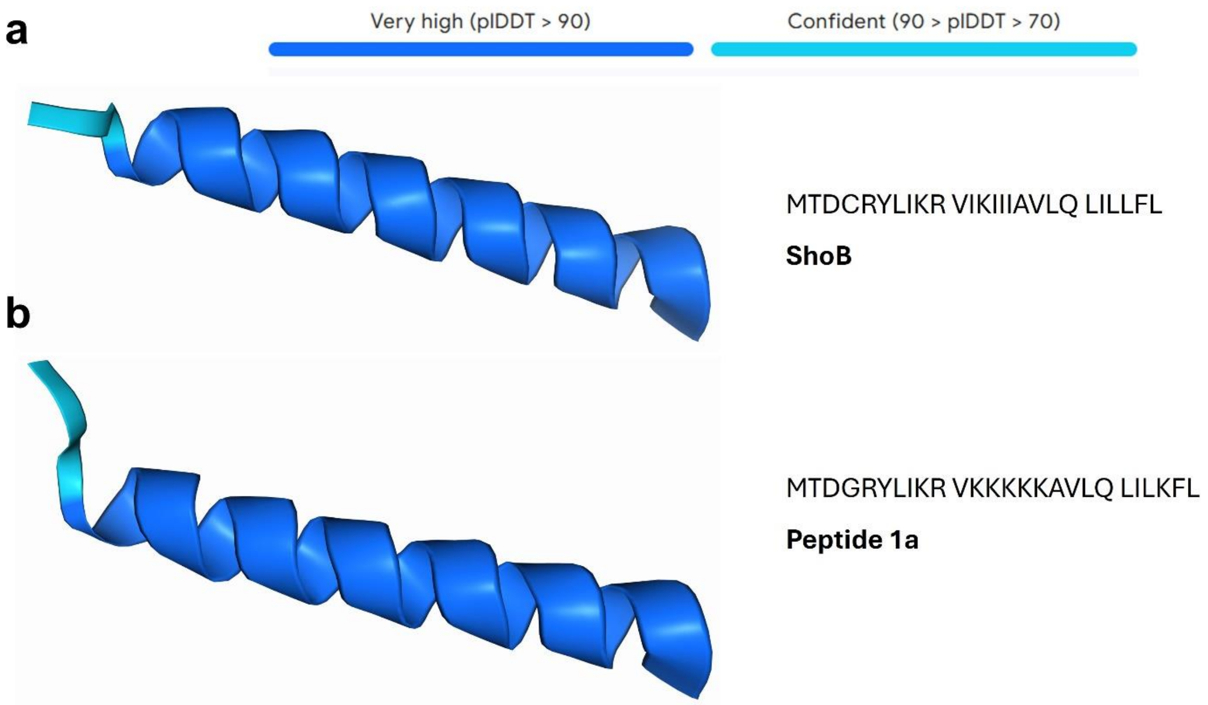

Figure S1: Structural prediction of ShoB and peptide 1a using AlphaFold. AlphaFold simulations predict α-helical conformations for both the native ShoB peptide (**a**) and modified peptide 1a (**b**). The predicted structures exhibit high confidence scores (pLDDT > 90) throughout most of their length, indicating reliable secondary structure predictions. The N-termini of both peptides show slightly lower confidence (90>pLDDT>70), suggesting some flexibility in these regions.

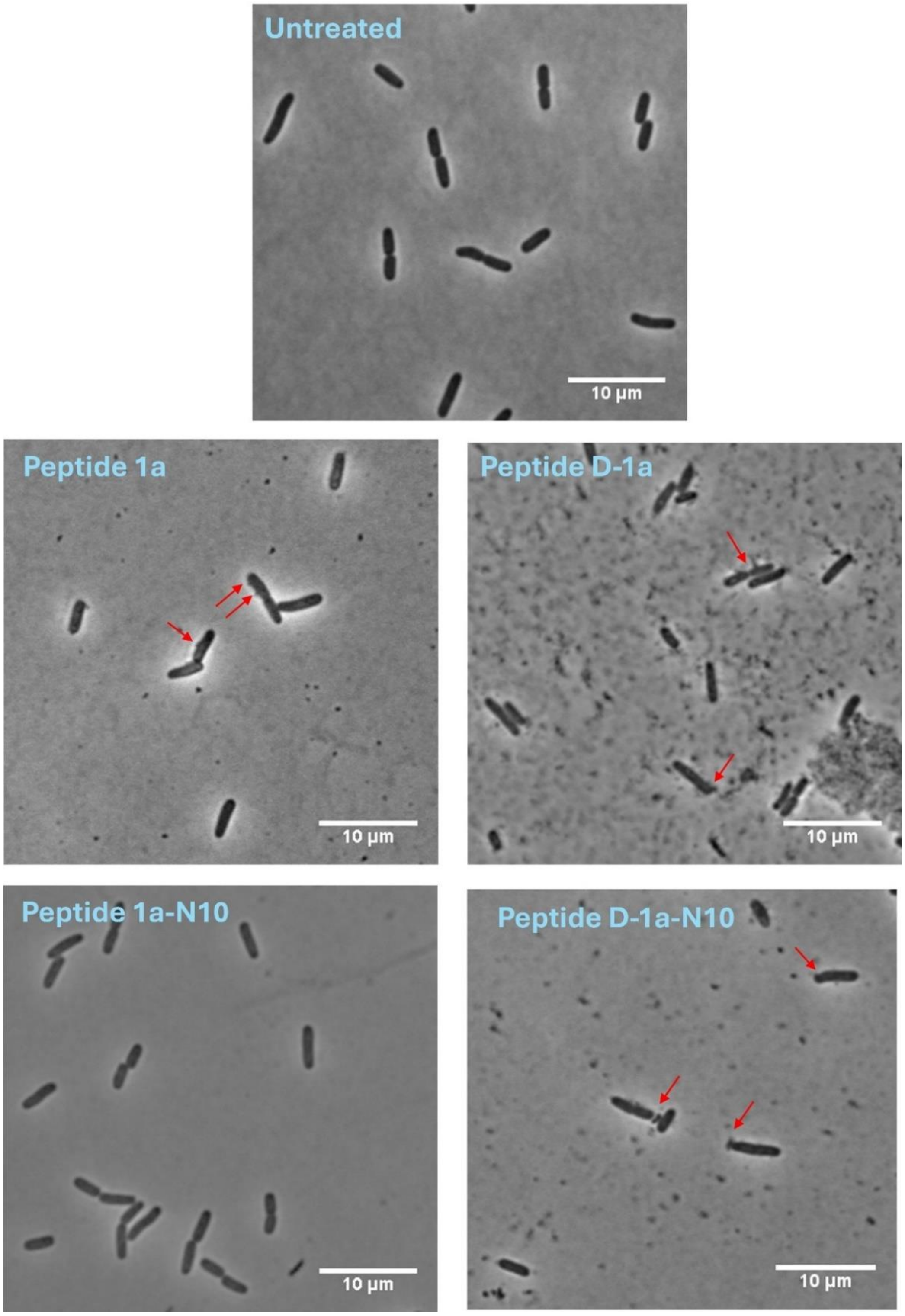

Figure S2: Morphological changes in *Escherichia coli* CCUG 17620 following antimicrobial peptide treatment. Representative phase contrast microscopy images of *E. coli* CCUG 17620 cells treated with peptides 1a, D-1a, 1a-N10, and D-1a-N10 at 10× their respective minimum inhibitory concentrations (MICs) compared to untreated control. Bacterial cultures were grown to an optical density (OD600) of 0.2, exposed to peptides for 5 minutes, and then immobilized on LB-agarose pads and covered with a #1.5 coverslip for imaging. Phase contrast imaging was performed using a Leica DM6000B microscope equipped with a HCX PLAPO 100×/1.40 NA objective. Imaging adjustments (brightness and contrast) were performed in Fiji software. Red arrows indicate clear alterations in cellular morphology.

Table S1: MIC and MBC bacterial panel assessment of peptide 2-N12 variants*.

ID	Sequence	MIC/MBC (μM)					
		E. coli	*S. aureus*	*K. pneumoniae*	*A. baumannii*	*P. aeruginosa*	*E. faecalis*
**2a-N12**	KKKKAVLQLI LLFL	>128/>128	>128/>128	>128/>128	>128/>128	>128/>128	>128/>128
**2b-N12**	KKKKIAVLQLI LLFL	>128/>128	>128/>128	>128/>128	>128/>128	>128/>128	>128/>128
**2c-N12**	KKKKIIAVLQL ILLFL	>128/>128	>128/>128	>128/>128	>128/>128	>128/>128	>128/>128
**2d-N12**	KKKKIIIAVLQ LILLFL	>128/>128	>128/>128	>128/>128	>128/>128	>128/>128	>128/>128
**2e-N12**	KKKK-FVLKLILKLL	>128/>128	>128/>128	>128/>128	>128/>128	>128/>128	>128/>128
**2f-N12**	KKKKFVLQLIL KLL	>128/>128	>128/>128	>128/>128	>128/>128	>128/>128	>128/>128
**2g-N12**	KKKKKKFVLK LILKLL	128/128	128/128	64/128	128/128	128/128	64/128
**2h-N12**	KKKKKKFVLQ LILKLL	>128/>128	>128/>128	>128/>128	>128/>128	>128/>128	>128/>128
**2i-N12**	RRRRFVLRLI LRLL	64/128	64/128	64/128	128/128	64/128	64/128
**2j-N12**	RRRRRRFVLR LILRLL	64/128	64/64	64/64	64/64	64/64	32/32

*Detection limit:<0.5 μM and >128 μM

Table S2: MIC and MBC bacterial panel assessment of peptide 3-N10 variants*.

ID	Sequence	MIC/MBC (μM)					
		E. coli	*S. aureus*	*K. pneumoniae*	*A. baumannii*	*P. aeruginosa*	*E. faecalis*
**3a-N10**	VRRRRRAVL QLILRFL	32/32	32/32	32/32	16/16	64/64	16/16
**3b-N10**	VKKKKKAVLQ LILKFLRRRR	16/16	8/8	16/16	8/8	4/4	4/4
**3c-N10**	VKKKKK-PEG2-AVLQLILKFL	16/16	32/32	16/16	16/16	64/32	16/16
**3d-N10**	VKK-PEG2-KKKAVLQLIL KFL	16/16	32/32	32/32	16/16	64/64	32/32
**3e-N10**	VKKKKKAVLQ LI-PEG2-LKFL	128/128	>128/>128	128/128	32/64	32/>128	>128/>128

*Detection limit:<0.5 μM and >128 μM
